# Detoxification-Assisted Bioprocessing for Sustainable Conversion of Defatted Spent Coffee Grounds to Bioplastics and Biofunctional Metallic Nanoparticles

**DOI:** 10.3390/polym18151846

**Published:** 2026-07-28

**Authors:** Ganesh Saratale, Ramesh Kumar, Han Seung Shin, Dong Su Kim, Rijuta Ganesh Saratale, Byong-Hun Jeon

**Affiliations:** 1 Department of Food Science and Biotechnology, Dongguk University-Seoul, Ilsandong-gu, Goyang-si 10326, Republic of Korea; ganeshfbtdongguk17@gmail.com (G.S.); spartan@dongguk.edu (H.S.S.); 2 Department of Earth Resources & Environmental Engineering, Hanyang University, Seoul 04763, Republic of Korea; rameshibt@gmail.com (R.K.); cherryg2310@gmail.com (R.G.S.); 3 Department of Environmental Science and Engineering, Ewha Womans University, Seoul 03760, Republic of Korea; dongsu@ewha.ac.kr

**Keywords:** defatted spent coffee grounds, *Lysinibacillus* sp. RG.S., polyhydroxyalkanoates, detoxification, silver nanoparticles, antioxidants, SCGO-AgNPs

## Abstract

Spent coffee grounds (SCGs) are waste products obtained after brewing roasted coffee beans. The detoxification-based process used in the preparation of defatted spent coffee grounds (SCGO) involves: (i) direct acid pretreatment followed by enzymatic-hydrolysis (AE), (ii) solvent extraction followed by acid pretreatment and enzyme-hydrolysis (SAE), and (iii) solvent extraction followed by acid pretreatment and enzymatic-hydrolysis coupled with activated carbon detoxification (SAEAc) for polyhydroxyalkanoates (PHA) production. SCGO was subjected to optimized acid pretreatment (100 °C, 2.0% H_2_SO_4_, 6 h), followed by enzyme saccharification (20 FPU/g of SCGO). The effect of detoxification on the obtained hydrolysates was comparatively evaluated based on their inhibitor concentrations and suitability as substrates for fermentation by *Lysinibacillus* sp. RG.S. and PHA biosynthesis. The maximum biomass productivity (5.6 ± 0.44 g/dm^3^), PHA accumulation (53.0 ± 1.22%), and PHA yield (2.97 ± 0.32 g/dm^3^) were produced by the hydrolysates that were generated by the SAEAc-detoxification process with 1% corn steep liquor (CSL) and 1% acetic acid. The physicochemical and thermo-stable properties of the produced PHA were found to be comparable to those of standard PHB, making it appealing for a variety of applications. Extracted bioactive compounds from SCGO were employed as reducing and stabilizing agents for the synthesis of silver nanoparticles (AgNPs). Analytical results suggest that the SCGO-AgNPs were well phyto-fabricated and evenly dispersed, with particle diameters of about 10–40 nm. Furthermore, SCGO-AgNPs demonstrated potential antioxidant and antidiabetic activities, indicating their prospective for biomedical and nutraceutical applications. The foregoing results illustrate that detoxification strategy enhances the fermentability of SCGO hydrolysate and extracted bioactive compounds are used for biofunctional synthesis of AgNPs. In summary, the integrated cascading biorefinery concept supports sustainable and economically viable valorization of SCGO.

## 1. Introduction

**Figure 1:**
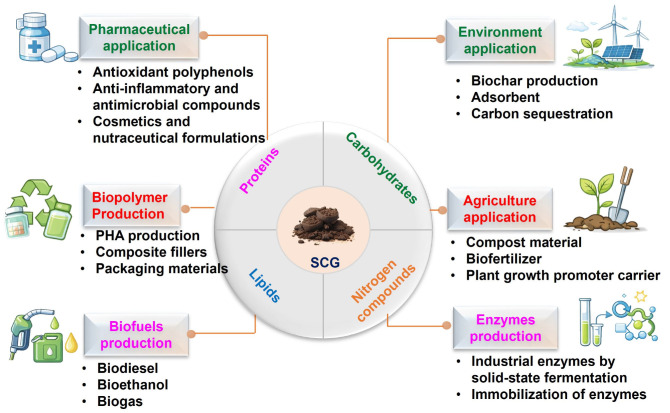
Chemical constituents of spent coffee grounds (SCGs) waste biomass and recent developments in their valorization into various high-value biocommodities.

Globally coffee is recognized as the most popular beverages consumed for over 1000 years and accounts for more than 400 billion cups per year [1,2]. It is also the world’s second-largest merchandised commodity after crude oil and its derivatives. The world production of coffee has increased by 59.3% over the past 20 years, and the resulting spent coffee grounds (SCGs) have become a serious environmental challenge [3]. The major solid by-product of roasted coffee beans is known as SCGs, which produce more than 0.65 kg of grounds per 1 kg of brewed coffee. Worldwide the production of SCGs is estimated to be 6–7 million tons per year [4,5]. Traditionally, SCGs have been disposed of in landfills or incinerated. Due to their high biodegradable organic matter concentration, landfilled SCGs are subjected to anaerobic digestion and are responsible for the release of greenhouse gases such as CH_4_ and CO_2_, thus increasing soil and air pollution [6,7]. Furthermore, soil contamination with SCGs can affect soil chemistry, induce phytotoxicity and microbial imbalance and lead to the release of organic pollutants that can adversely affect environmental quality [8,9]. The incorporation of SCGs is generally acidic (pH ~4.5–6.0), which can increase soil acidity and support acid-tolerant microorganisms, but also inhibit species that require higher pH levels, thus changing the biological diversity and nutrient cycling of the soil. Despite such concerns, SCGs have come into focus as a valuable and renewable biomass resource owing to their high chemical compositions. SCGs are composed of cellulose, hemicellulose, lignin, lipids (10–20%), proteins, minerals, polyphenols and other bioactive components. Development in the valorization of SCGs has shown great potential for biofuels, bioplastics, biochar, adsorbents, agricultural amendments, nutraceuticals, functional nanomaterials, and platform chemicals and other high-value biomaterials (Figure 1). Therefore, SCGs are increasingly regarded in the context of a circular economy as a viable feedstock to be utilized for resource recovery and integrated biorefinery processes [10,11,12].

The global bioplastics market offers immense growth opportunities to generate sustainable products that can be utilized in different forms [13,14]. The world capacity of bioplastics reached 2.31 million tons (Tg) in 2025. Polyhydroxyalkanoate (PHA) can be completely biodegraded, and the final products consist of non-toxic products, thus making it environmentally friendly [15,16]. PHA have been produced on industrial scales of 1000–10,000 tons (Tg) per year in major companies around the world, such as Biomer (Germany), Bio-On (Italy), CJ Bio (Republic of Korea), Danimer Scientific (USA), TianAn Biologic Materials Co. (China), and RWDC Industries Ltd. (Singapore) [17]. However, high production costs, the use of pure substrates and low efficiency when using agro-industrial waste limit the scale at which the PHA can be bio-produced [18,19]. After extracting oil from SCGs the residual defatted biomass (denoted as SCGO) can serve as boiler fuel, partially supplying energy for extraction, sterilization, and fermentation. It can be utilized for biorefinery applications [5]. The valorization of SCGO as a feedstock to produce PHA is a promising strategy to decrease the cost of production and enhance environmental sustainability. This concept has been extensively explored in this research work.

However, the use of SCGO for microbial fermentations is limited due to the presence of high quantities of phenolics such as catechin, chlorogenic acid, caffeic acid, ferulic acid, gallic acid, ellagic acid, *p*-hydroxybenzoic acid, *p*-coumaric acid, rutin, protocatechuic acid, quercetin and tannic acid. These bioactive compounds have antioxidant and, antimicrobial properties and may interfere with the growth of PHA-producing bacteria [3,7]. Thus, necessitating the removal and/or extraction of these compounds before SCGO can be valorized into high-value bio-products. In addition, SCGO is a recalcitrant lignocellulosic biomass with a complex, rigid structure, which creates hurdles for enzyme hydrolysis [20]. Effective pretreatment techniques should be developed to increase the enzymatic accessibility of the biomass and to enhance the release of fermentable sugars. Various biological, chemical, physical, and physicochemical pretreatment methods have been investigated to remove lignin, followed by enzymatic saccharification to release fermentable sugars [21].

Acid pretreatment is important for disrupting the rigid lignocellulosic structure, hydrolyzing hemicellulose, breaking lignin-carbohydrate linkages, and increasing cellulose accessibility. This structural modification increases enzyme uptake, facilitates the release of fermentable sugars and improves the efficiency of consequent fermentation processes [22,23]. During acid hydrolysis of biomass, the degradation of pentose and hexose sugars, generates inhibitory by-products, primarily furfural (2-furaldehyde) and 5-hydroxymethylfurfural (5-HMF). Even at relatively low concentrations (<0.1 g/L), these compounds can adversely affect microbial metabolism and substantially suppress the growth of many microorganisms by disrupting the cell walls, intracellular pH, respiration, and/or cell replication [24]. The combined adverse impacts of all toxic compounds in their lignocellulosic hydrolysates are found to be several times higher than the adverse impact of the individual compounds [25,26].

Various detoxification treatment technologies have been investigated for the removal or reduction in inhibitory compounds, such as overliming, activated charcoal adsorption, ion exchange resins, solvent extraction, biological scrubbing and vacuum evaporation [27]. The effectiveness of these methods is greatly reliant upon the type and concentration of inhibitors and the characteristics of the microbial strain used [26]. Hence, it is crucial to optimize detoxification strategies to maximize the microbial vitality, enhance PHA accumulation, and ensure sustainable conversion of SCGO waste into valuable bioplastics during the integrated biorefining process. Nanotechnology, an emerging field, has developed to design and employ resources at the nanoscale level, unlocking exceptional functionalities and properties [28,29]. Scientists have been concentrating on using the biological approach to synthesize nanoparticles because they are normally non-toxic, environmentally benign, and economical [30,31,32]. Silver nanoparticles (AgNPs) were synthesized using the extracted bioactive compounds from SCGO as reducing, capping, and stabilizing agents.

The aims of this study are to compare distinct routes to SCGO hydrolysate preparation, to evaluate the effectiveness of detoxification in removing inhibitory compounds, and to assess the effect of these hydrolysates on the growth and production of PHA by *Lysinibacillus* sp. RG.S. Furthermore, optimization of fermentation conditions and characterization of the manufactured PHA will be studied. To investigate the potential of using solvent-extracted bioactive compounds from SCGO for green synthesis of silver nanoparticles and examine their functional properties. This provides a beneficial scope towards the sustainable cascading biorefinery concept in a circular bioeconomy.

## 2. Materials and Methods

### 2.1. Handling of SCGO Feedstock—Collection and Preparation

Fresh spent coffee grounds (SCGs) were collected from a local coffee shop in Goyang-si, Republic of Korea (approximately, 37.6584° N, 126.8320° E) and kept in the freezer at −20 °C until further use. The material was dried for 24 h in an oven at 105 °C until a constant weight was achieved before extraction was undertaken to remove moisture. The Soxhlet extraction method was used to extract lipids from SCGs biomass using 200 mL of *n*-hexane and refluxing for 2 h at 70 °C. Crude coffee oil was obtained from the solvent phase and the lipid extract was concentrated using a rotary evaporator at 50 °C and 50 rpm for 30 min. The extracted oil recovered was gathered, and the extraction yield was measured gravimetrically against the initial dry mass of SCGs with the following equation.

(1) $$SCGs\;oil\;extraction\;yield\;\left(\%\right)=\frac{Initial\;dry\;SCG\;mass-Final\;defatted\;SCG\;mass\;}{Initial\;dry\;SCG\;mass}\times 100$$

The spent solid residue after extraction was removed and dried under a fume hood for 24 h to remove the residual solvent, and the defatted biomass was determined by the gravimetric method. No additional washing step was performed after the extraction. The defatted spent coffee ground biomass (SCGO) was dried and kept in air-tight plastic bags in a refrigerated area until further processing. Double-distilled water (Millipore Co., Billerica, MA, USA) was used during the preparation of all aqueous solutions. Tryptic Soy with no dextrose obtained from Becton Dickinson, Franklin Lakes, NJ, USA. Yeast extract and peptone were purchased from Becton Dickinson (Sparks, MD, USA). Chloroform, sodium hypochlorite, sodium citrate, d-glucose, NaH_2_PO_4_, Na_2_HPO_4_, K_2_SO_4_, MgSO_4_·7H_2_O, CaCl_2_, MnSO_4_·H_2_O and (NH_4_)_2_SO_4_, H_2_SO_4_, Whatman filter paper No. 1, activated charcoal were obtained from Dae-Jung chemicals, Siheung-si, Republic of Korea. Silver nitrate (AgNO_3_), ethanol, ascorbic acid, 2,2′-Azino-bis(3-ethylbenzothiazoline)-6 sulfonic acid (ABTS), Folin–Ciocalteu’s phenol reagent (2 N), 2,2-diphenyl-1-picryhydrazyl (DPPH), 3,5-dinitrosalicylic acid (DNS), corn steep liquor, sodium potassium tartrate, α-glucosidase, acarbose, and α-amylase were all purchased from Sigma-Aldrich Chemicals (St Louis, MO, USA). All chemicals used for oil extraction, chemical pretreatment and the production and recovery of polyhydroxyalkanoate (PHA) for bioactivity assays were of analytical grade and high purity.

### 2.2. Preparation of SCGO Feedstock by Detoxification Strategies

**Figure 2:**
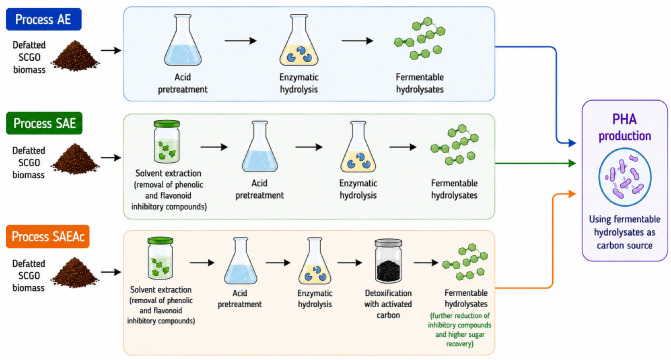
Schematic illustration of detoxification routes used in the preparation of SCGO feedstock for the production of PHA by *Lysinibacillus* sp. RG.S.

Three detoxification treatments were attempted on defatted spent coffee grounds (SCGO) to convert them into fermentable hydrolysates for the production of polyhydroxyalkanoate (PHA) (Figure 2).

(i) In the process AE, biomass was treated directly with acid and then subjected to enzymatic hydrolysis.
(ii) In the process SAE, there was a preliminary step of solvent extraction to remove phenolic and flavonoid inhibitory compounds, followed by acid pretreatment and enzymatic hydrolysis.
(iii) In the process SAEAc, solvent extraction followed by acid pretreatment and enzymatic hydrolysis, coupled with an additional detoxification step using activated charcoal.

Solvent extraction of SCGO for the removal of phenolics and flavonoids was performed. In total, 10 g of SCGO was extracted with 50 mL of 80% methanol using constant stirring (150 rpm, in the dark) for 3 h at 65 °C. Following the extraction process, the mixture was filtered through Whatman No. 91 filter paper. Using a rotary evaporator, the obtained filtrates were concentrated under reduced pressure, after which the solvent was evaporated to produce the dry extract for characterization.

The residual biomass after extraction was filtered, dried, and further tested for chemical pretreatment. For each detoxification process, SCGO undergoes optimized acid hydrolysis pretreatment (2.0% H_2_SO_4_, 100 °C, 6 h) and is then enzymatically saccharified. The ratio of solid to liquid was 1:10 for each treatment. After pretreatment, the mixtures were allowed to centrifuge at 4000 rpm for 20 min (Labogene 1736R, Lillerod, Denmark) to separate the solid fraction. The pretreated SCGO biomass was then thoroughly washed with distilled water until a neutral pH (pH 7.0) was achieved and oven dried at 60 °C until a constant biomass weight was achieved. Dried pretreated SCGO samples were employed for saccharification using the crude enzyme cocktail in the amount of 20 FPU/g of SCGO prepared by *Streptomyces* sp. MDS. The enzymes were produced via solid-state fermentation of wheat straw under optimized conditions as described in an earlier study [33]. The activities of the major cellulolytic enzymes, including endoglucanase 12.5 U/mL, exoglucanase 1.75 U/mL, and β-glucosidase10.2 U/mL are present. The liquid hydrolysate was then separated and detoxified using activated charcoal treatment, which removes fermentation inhibitors such as furfural and 5-hydroxymethylfurfural (HMF). Activated charcoal was added at 25 g/L and the mixture was agitated at 150 rpm and 45 °C for 60 min to promote the adsorption of inhibitory compounds. The adsorption process parameters were selected based on our preliminary laboratory experiments these conditions provided good adsorption efficiency. The charcoal was then separated by centrifugation at 1100× *g* for 15 min [34]. The concentrations of reducing sugars and inhibitory compounds were measured before and after detoxification to evaluate the effectiveness of the treatment.

### 2.3. PHA Production Studies from Detoxified SCGO Feedstock

The PHA production ability of an isolated strain of *Lysinibacillus* sp. RG.S. was assessed and compared with three detoxified enzymatic hydrolysates (AE, SAE, and SAEAc) obtained from SCGO. For PHA biosynthesis, 5% (*v*/*v*) bacterial inoculum was transferred into a production medium comprising the following mineral salts (g/dm^3^): NaH_2_PO_4_, 3.6; K_2_SO_4_, 3.486; Na_2_HPO_4_, 2.84; NaOH, 0.4; MgSO_4_·7H_2_O, 0.39; (NH_4_)_2_SO_4_, 0.1; yeast extract, 0.2; CaCl_2_, 0.062; MnSO_4_·H_2_O, 0.024; CuSO_4_·5H_2_O, 0.005; FeSO_4_·7H_2_O, 0.15; and ZnSO_4_·7H_2_O, 0.024. The initial medium pH was fixed at 7.0, and each detoxified SCGO hydrolysate was supplemented to achieve an equivalent sugar concentration of approximately 20 g/dm^3^. Batch fermentations using a production medium (100 mL) were conducted. The cultivation parameters of the experiments were optimized to maximize PHA production from the SCGO hydrolysates at 30 °C, pH 7.0, agitation at 200 rpm and a fermentation time of 72 h. These optimized operating conditions were subsequently maintained throughout all PHA production experiments.

### 2.4. Supplementation of Nutrient and External Stress Conditions to Increase PHA Production

The SAEAc detoxified SCGO hydrolysate was chosen as the substrate for the study of methods to strengthen the growth of the bacteria and the production of PHA. The nutrient supplement effects were tested by adding optimized levels of complex nutrient sources (corn steep liquor CSL (1% *v*/*v*) and peptone (1% *w*/*v*) into the fermentation media). Furthermore, the effect of external metabolic stresses on the PHA production was investigated by adding NaCl (10 g/dm^3^) and acetate (1% *v*/*v*) to the culture medium. Different media formulations of CSL (1% *v*/*v*), acetate (1% *v*/*v*), and SAEAc-detoxified SCGO hydrolysate (about 20 g/dm^3^ fermentable sugars) were tested for their significance on the growth of cell and PHA production capability of *Lysinibacillus* sp. RG.S. The bacteria growth-associated kinetic parameters and PHA production parameters were calculated according to the equations described previously [18]. Each experiment was conducted three times, and the average of the three experimental runs is shown.

### 2.5. PHA Extraction and Purification

After 72 h of growth, the bacterial biomass was retrieved with centrifugation and then freeze-dried. A solvent-assisted recovery method was used to extract PHA from lyophilized cells. In summary, dried biomass was mechanically homogenized in chloroform, to dissolve the intracellular polymer. The suspension was subsequently treated with sodium hypochlorite, which breaks cells open to recover the purified polymer from them, effectively removing nucleic acids, proteins, and membrane components of the cells, which are not polymeric, from the recovered polymer. Polymer was precipitated with 80% (*v*/*v*) methanol as described previously and recovered by vacuum filtering [35]. Additionally, to increase polymer purity, the recovered PHA was re-precipitated with 80% (*v*/*v*) methanol, vacuum filtered and washed with methanol–chloroform to remove impurities. The purified polymer was then placed in a hot air oven to dry for 48 h at 60 °C to reach constant weight [18]. The purified PHA was then subjected to physicochemical characterization using various analytical techniques.

### 2.6. Phytochemical Characterization of Solvent Extractives from SCGO

The solvent extractives of SCGO were qualitatively screened to determine major bioactive constituents, such as the tannins, alkaloids, terpenoids, flavonoids, saponins, steroids, glycosides, phenolics, anthraquinones and cardiac glycosides, using standard protocols [36]. To quantify the total phenolic content of the extracts, the Folin–Ciocalteu colorimetric method was used [37]. The total phenolic content (TPC) was expressed as mg gallic acid equivalents (GAE) per gram of dry extract (DW). The total flavonoid content (TFC) was estimated by the aluminum chloride colorimetric method reported by Kumaran and Joel (2007) [38] using rutin as a standard. In brief, 1 mL of the extract solution (SCGO extractives 10 mg/mL in methanol) was added to 1 mL of aluminum trichloride (AlCl_3_) solution (20 mg/mL in ethanol), and a drop of glacial acetic acid (97–99% purity) was added. The reaction mixture was incubated at room temperature for 40 min, then diluted with 25 mL of ethanol. The absorbance was measured at 415 nm spectrophotometrically. Flavonoids were determined as milligrams of rutin equivalent (RE) per gram of dry extract.

### 2.7. Silver Nanoparticles (AgNPs) Were Synthesized Using Extractives from SCGO

Silver nanoparticles (AgNPs) were prepared through a “green” chemical method using SCGO extractives. Briefly, SCGO extract was mixed with silver nitrate (AgNO_3_) solution (1 mM) with continuous stirring in an Erlenmeyer flask. The reaction mixture was further incubated at 40 °C, which was suitable for reducing Ag^+^ ions and promoting the formation of AgNPs. Initially, a remarkable color change from a pale yellow to dark reddish-brown of the reaction mixture, suggesting the reduction of silver ions by the bioactive phyto-constituents present in the SCGO extract. Under optimum conditions (temperature 40 °C; mixing ratio SCGO extract to AgNO_3_ (1:10)), nanoparticles formation occurred completely within 20 min; the reduction was monitored by UV–visible spectrophotometry. After synthesis, the solution was centrifuged at 12,000 rpm for 15 min to recover the AgNPs. The unbound biomolecules and impurities were removed by washing the nanoparticle pellet several times. The purified AgNPs were then dried in a hot-air oven at 60 °C for 24 h [32] and kept for subsequent analytical characterization and assessment of their biological activity.

### 2.8. Antioxidant and Antidiabetic Potential of Biosynthesized SCGO-AgNPs

The antioxidant activity of the biosynthesized SCGO-AgNPs was investigated with two popular and widely used radical scavenging assays namely 2,2-diphenyl-1-picrylhydrazyl (DPPH), 2,2′-azino-bis-(3-ethylbenzothiazoline-6-sulfonic acid) (ABTS) assays. In the ABTS assay, the concentration of the SCGO-AgNPs lies between 25 and 100 μg/mL. In brief, 2 mL of each nanoparticle suspension was added to freshly prepared ABTS•^+^ solution, followed by 10 s of vortexing and 6 min of incubation at room temperature. The ABTS radical cation was allowed to undergo reduction by the antioxidant molecules, thereby decreasing the absorbance, and was measured spectrophotometrically at 734 nm [32]. SCGO-AgNPs were evaluated for their DPPH radical scavenging activity using the standard protocol where catechol was used as the reference antioxidant. Antioxidant activity was monitored by the reduction in the DPPH radical, shown by the characteristic color change from deep violet to pale-yellow measured at 517 nm [32]. The percentage radical scavenging activity was calculated with respect to two radical scavenging assays, DPPH and ABTS, using the following equation:

(2) $$\%\;Scavenging\;=\frac{Cabs-Tabs}{Cabs}\times \;100$$

C_abs_ is for the absorbance of the control, and T_abs_ is the absorbance of the SCGO-AgNPs sample.

α-glucosidase inhibition assay was assessed to evaluate the antidiabetic activity of the SCGO-AgNPs. Briefly, the SCGO-AgNPs (25–100 μg/mL) were incubated with the sodium phosphate buffer (0.1 M, pH 6.9) at 37 °C for 10 min, then supplemented with 2 mM *p*-nitrophenyl-α-D-glucopyranoside and incubated for another 20 min at 37 °C. The reaction was stopped by adding 0.1 M Na_2_CO_3_, and the absorbance was determined at 405 nm [39]. The α-amylase inhibitory activity of SCGO-AgNPs was determined using the standard protocol illustrated earlier [32]. For both assays, acarbose acts as the positive control. The inhibition in percentage is calculated using the equation below:

(3) $$Inhibitory\;enzyme\;activity\;(\%)=\frac{\left[Ac-At\right]}{Ac}\times 100$$

where absorbance of the control is ‘Ac’ and absorbance of the sample with SCGO-AgNPs is ‘At’.

Each enzyme assay was performed in three replicates, and the results are shown as mean ± standard deviation. Dose–response curves were used to determine IC_50_ values and to compare the antioxidant, and antidiabetic inhibitory activities of SCGO-AgNPs.

### 2.9. Analytical Methods

Spent coffee grounds were analyzed for their cellulose, hemicellulose and lignin contents using the [40]. Goering and Van Soest method. The presence of fermentable sugars and other soluble metabolites in SCGO hydrolysates was quantified utilizing HPLC (Agilent model 1200, Palo Alto, CA, USA) with a SH1011 column (Shodex, Tokyo, Japan) capable of the refraction index detection. The mobile phase was 10 mM H_2_SO_4_ at 0.5 mL/min and the column temperature was kept at 55 °C [35]. Content of starch was assessed using the kit of Megazyme Total Starch Assay Kit (Amyloglucosidase/α-amylase method; K-TSTA-50A/K-TSTA-100A Wicklow, Ireland) using both the AOAC Method 996.11 and the AACC Method 76–13.01. The content of total nitrogen was assessed using an elemental analyzer (EA 1112, Thermo Finnigan, Somerset, NJ, USA) and protein content was determined as nitrogen multiplied by a coefficient of 6.25. The dinitrosalicylic acid (DNS) method of Miller [41]. was used to measure total reducing sugars. The total cellulase activity was determined by the IUPAC protocol [42] in filter paper units (FPU), defined as the amount of cellulase required to liberate 1 mmol of reducing sugars per minute under the specified test conditions. Chemical structures of the SCGO extract, prepared SCGO-AgNPs and synthesized PHA were characterized using Fourier-transform infrared (FTIR) spectroscopy (Cary 630, Agilent Technologies, Santa Clara, CA, USA) over the spectral range of 4000–400 cm^−1^ and 4 cm^−1^ resolution.

X-ray Diffraction (XRD) with a D2 Bruker diffractometer (Bruker, Berlin, Germany) which operates at 30 kV and 20 mA with Cu Kα radiation (λ = 1.5406 Å) to determine the crystal structure of the produced PHA. A 2θ range of 10 to 60° was scanned at room temperature with a diffraction rate of 1°/min. Thermal stability of PHA was assessed using thermo-gravimetric analysis (TGA) by a thermo-gravimetric analyzer (TGA Hi-Res 2950, TA Instruments, New Castle, DE, USA). About 10 mg of the sample was subjected to heat between 20 and 800 °C at a rate of 20 °C per minute under a N_2_ atmosphere. The thermal properties, such as melting point (*T_m_*) and glass transition temperature (*T_g_*) were estimated by a differential scanning calorimeter (TA Instruments, DSC2920, New Castle, DE, USA). About 2 mg of PHA was placed in aluminum pan and subjected to heating-cooling cycles of −50 to 250 °C at a heating and cooling rate of 10 °C and 5 °C per minute, respectively. Experiments for TGA and DSC were carried out as previously studied [36]. High resolution transmission electron microscopy (HR-TEM) images of the organized SCGO-AgNPs have been obtained to investigate the exterior morphology, exterior sizes, and the allocation of AgNPs using a Tecnai G2 20 S-TWIN (FEI Company, Hillsboro, OR, USA) machine. Distribution size and number of NPs were measured by manually determining the median diameter of 100 individual NPs in TEM images.

### 2.10. Statistical Analysis

Analysis of statistical experimental data was subjected to a one-way ANOVA. After ANOVA, Tukey’s honestly significant difference (HSD) test was carried out to determine statistically significant differences in mean values using Graph Pad Prism InStat software (version 3.06; GraphPad Software Inc., San Diego, CA, USA). The level of significance used was *p* < 0.05 in all statistical analyses.

## 3. Results and Discussion

### 3.1. Chemical Composition of SCGs

Various parameters like coffee bean origin, processing and roasting conditions strongly affect the chemical composition of spent coffee grounds (SCGs) [43,44]. The monosaccharide composition of the raw SCGs used in this study showed that 49.4% (*w*/*w*) of total sugars consisted of mannose (17.2%), galactose (20.2%), glucose (6.35%), cellobiose (2.60%), xylose (2.25%), and arabinose (0.8%) (Table 1). The content of this carbohydrate fraction was about 9% cellulose and 40% hemicellulose. Besides carbohydrates, the SCGs also contained 21.0% lignin, 11.5% extractable oil and 8.5% protein. In addition, the remaining fraction may include ash and other minor components that were not determined. Determination of biomass composition gives a valuable basis for studies of the effectiveness of pretreatment processes and enzymatic hydrolysis processes. The lignin content of the SCGs was found to be slightly lower than reported in some previous studies, while the total carbohydrate content was in line with results reported elsewhere [45,46,47,48]. In general, the chemical analysis results in this study highlighted that the SCGs are a carbohydrate-rich lignocellulosic residue that has high potential to produce value-added bioproducts and bio-based chemicals.

### 3.2. Detoxification Strategies and Effects on the Chemical Composition of SCGO Feedstock

The detoxification of SCGO biomass is a crucial step since this biomass contains a high concentration of phenolic and other bioactive compounds that tend to inhibit microbial growth and consequently have an adverse impact on the fermentation process. Thus, these inhibitory compounds are eliminated by solvent extraction before the acid pretreatment to improve the bioconversion efficiency of SCGO biomass. Phenolic compounds play an important role as plant secondary metabolites, exerting significant effects on plants under exposure to environmental stresses such as UV radiation and pathogen attack [49]. The high redox properties make them potent antioxidants, and they are found to be effective at binding free radicals, chelating metal ions, and neutralizing ROS that cause oxidative damage [50]. The TPC of solvent extracted SCGO was about 2.15 ± 0.01 (mg of GAE/g of SCGO) using methanol, which was investigated. This result aligns with earlier research, which showed that highly polar solvents such as methanol and ethanol were more efficient for extracting phenolic compounds from lignocellulosic biomass due to their solvation power and affinity for polar phytochemicals [51]. Flavonoids are a pivotal group of polyphenolic compounds with antioxidant and bioactive properties. The TFC obtained in the methanol extract provided the maximum amount of TFC (1.15 mg QE/g of SCGO). Efficient methanol extraction of flavonoids from coffee waste is evidenced by similar findings for spent coffee grounds and other coffee-based biomasses [50,52]. The results show that the solvent extraction (methanol) procedure was able to decrease the amount of phenolics present in the SCGO biomass while recovering some other valuable antioxidant phytochemicals.

**Figure 3:**
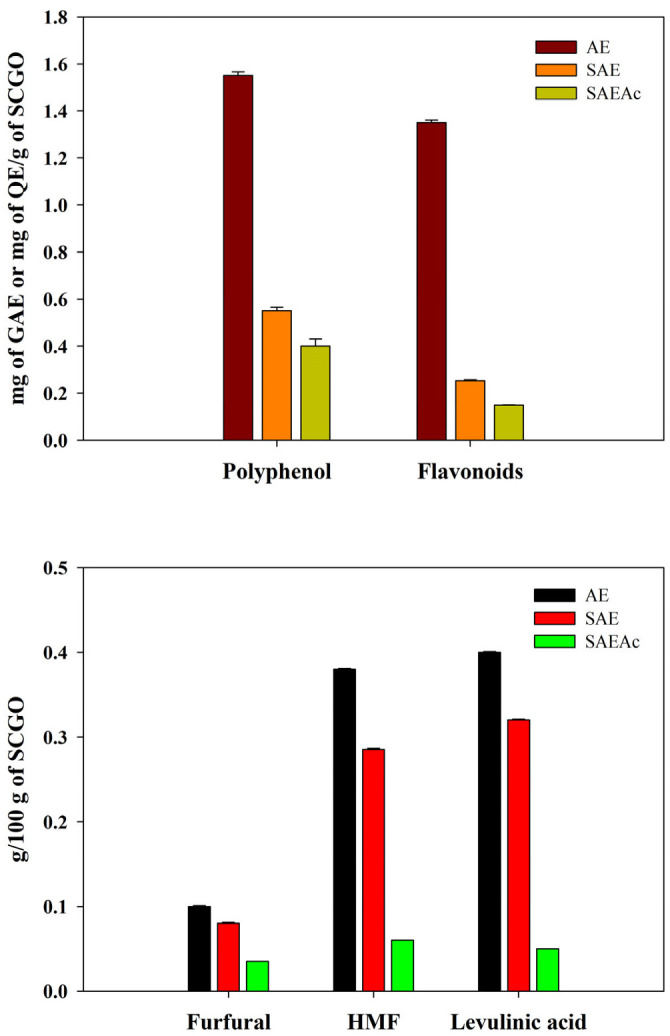
Concentrations of polyphenolic compounds (mg of GAE/g of SCGO), flavonoid content (mg of QE/g of SCGO), and acidic and furan derivatives were generated during the proposed detoxification strategies of SCGO feedstock.

The fermentable monosaccharides generated by enzymatic saccharification are a promising feedstock for the microbial production of PHAs. Linking PHA biosynthesis to the valorization of SCGO could open the way to convert low value residues into high-value substances, enhancing the techno-economic potential of an integrated biorefinery process [53]. A sustainable cascade valorization strategy was developed to exploit SCGO using a sequential recovery of high-value products. In the first part, bioactive phenolic compounds were harvested and used as reducing and stabilizing agents for the green synthesis of functional silver nanoparticles (AgNPs). The phenolic-depleted solid residue was then subjected to acid pretreatment followed by enzymatic saccharification to release fermentable sugars. The enzymatic hydrolysates obtained were then explored for their potential use as a renewable feedstock for microbial production of the polyhydroxyalkanoate (PHA) biopolymer, creating an integrated waste-to-biopolymer platform. Although acid pretreatment was effective for biomass deconstruction, it also produced inhibitory compounds, such as organic acids and furan derivatives including furfural and 5-hydroxymethylfurfural (HMF), which can inhibit microbial growth and/or enzymatic activity during fermentation [54,55]. Hence, an attempt was made to detoxify the hydrolysate with activated charcoal which has a higher surface area and porosity, making it efficient in adsorbing the inhibitory molecules. These compounds can be removed to enhance the quality of the hydrolysate, acceptability to microorganisms and, ultimately, the efficiency of PHA production. Thus, hydrolysate detoxification is a key prerequisite for the effective bioconversion of SCG-derived sugars into value-added bioproducts. Concentrations of major inhibitory compounds, such as phenolic compounds, flavonoids and furan derivatives such as furfural and 5-hydroxymethylfurfural (HMF), were quantified systematically throughout the proposed three-stage detoxification strategy (Figure 3). The results showed significant differences among the three detoxification methods, with the least inhibitory compounds recorded in the SAEAc method. The efficient removal of both phytochemical inhibitors and those produced by the pretreatment both led to a higher-quality hydrolysate suitable for microbial fermentation. The enzymatic hydrolysates were then used as carbon sources for the production of PHA using *Lysinibacillus* sp. RG.S. for the evaluation of its bioconversion potential.

### 3.3. PHA Production from SCGO-Derived Hydrolysates

SCGO hydrolysate mainly contains mannose, galactose, and glucose, sugars that can be used as substrates for microbial polyhydroxyalkanoate (PHA) biosynthesis. However, the application of SCGs hydrolysates in the production of PHAs is not yet well explored. Native bacterial strains have been used to produce PHA from SCG-derived hydrolysates. *Burkholderia cepacia* CCM 2656, grown in a chemo-enzymatic SCG hydrolysate (7.52 g/dm^3^ sugars), accumulated 25.4% (*w*/*w*) PHBV over a period of 72 h, with a yield coefficient (*Y_p_*_/*s*_) equal to 0.10 [7]. Similarly, *Halomonas halophila* CCM 3662 produced 15.9% (*w*/*w*) PHA (0.35 g/L) from acid-hydrolyzed SCG hydrolysates after 72 h of cultivation [26]. PHA production from SCG hydrolysates has previously been achieved with microorganisms such as *Pseudomonas fluorescens*, *Bacillus subtilis*, *B. firmus*, and *Burkholderia cepacia*, despite their good ability to metabolize these hydrolysates, PHA titers and productivities were generally modest [26,56]. The results indicate that the SCGs can be considered as a sustainable feedstock for microbial PHA production.

**Figure 4:**
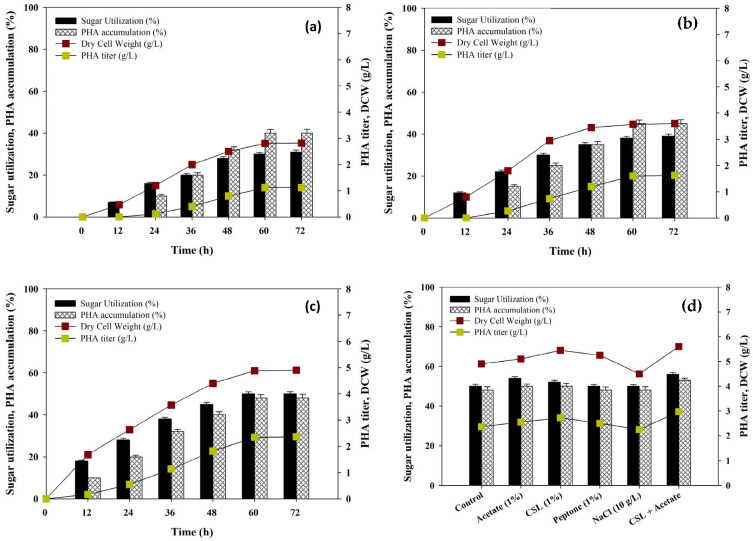
(**a**–**c**) Effects of detoxification strategies in the preparation of SCGO hydrolysates (20 g/dm^3^) and (**d**) supplementation of nutrients on assimilation of sugar, *Lysinibacillus* sp. RG.S. growth and PHA production parameters.

*Lysinibacillus* sp. RG.S. has been previously explored for the degradation of environmental pollutants and biotransformation of different waste streams [57]. The isolated bacteria *Lysinibacillus* sp. RG.S. has demonstrated substantial production of PHA across a wide range of detoxified and non-detoxified lignocellulosic hydrolysates. Accordingly, its capacity to convert SCGO hydrolysates into PHAs is investigated in the present study. The used detoxification strategies showed significant effects on the bacteria growth and PHA production efficiency. Direct acid pretreatment and enzymatic hydrolysis led to hydrolysates with higher levels of phenolics, flavonoids, furfural, and 5-hydroxymethylfurfural (HMF) and resulted in lower saccharification and fermentation yields by the microbes. As a result, ~30% of sugars were utilized, leading to a moderate bacterial growth and PHA yield of 1.12 g/dm^3^ (Figure 4a). In addition, only the polyphenol removal step is not enough to remove inhibition to microbial-PHA production since large amounts of furan derivatives and other toxic compounds were present in the hydrolysate. Due to this, the SAE detoxification strategy turned out ineffective for sugar consumption (about 38%) and PHA production (about 1.60 g/dm^3^), respectively (Figure 4b).

The SAEAc detoxification method showed the most beneficial treatment performance. Hydrolysates prepared by this method showed better fermentability resulting in about 50% sugar use, biomass yield to 4.90 g/dm^3^ and the maximum production of PHA reached 2.36 g/dm^3^ (Figure 4c). The enhanced performance is probably due to the successful removal of furanic inhibitors noted in SCGO hydrolysates by activated charcoal (Figure 3), which is well established as one of the most effective treatments for detoxification of lignocellulosic hydrolysates. Similarly, in the case of *Burkholderia cepacia*, *Burkholderia sacchari*, and *Cupriavidus necator*, the activated charcoal treatment resulted in significantly lower concentration of inhibitory compounds in the biomass hydrolysates, leading to better microbial growth and PHA productivity [26,58,59]. To further enhance PHA production from SCGO hydrolysates, in the present study, supplementation of SAEAc hydrolysates with 1% corn steep liquor (CSL) and 1% acetic acid resulted in the highest biomass concentration (5.62 g/dm^3^), PHA accumulation (53.0%), and PHA titer (2.97 g/dm^3^) (Figure 4d, Table 2).

Despite these improvements, complete utilization of SCGO-derived sugars was not achieved. Carbohydrate composition of the SAEAc hydrolysate was analyzed by HPLC before and after fermentation. Glucose (48.5%) was the major sugar, followed by galactose (24.8%), mannose (19.5%) and xylose (5.5%) before fermentation, and no cellobiose or arabinose were found. Following fermentation, the glucose concentration was completely utilized, the xylose concentration was reduced to 2.2%, the mannose concentration was reduced to 17.5%, and the galactose did not change. The following sugars were not detected: Cellobiose and arabinose. These results suggest that *Lysinibacillus* sp. RG.S. prefers to metabolize certain carbohydrate fractions, especially the glucose component derived from cellulose, and has a low capacity to use others (such as mannose and galactose). In addition, it is likely that there exist some compounds in the hydrolysates that inhibit the bacteria and colored contaminants that persists limiting the activity of the bacteria. Process performance may also be significantly boosted through the application of metabolic engineering to optimize galactose and mannose utilization effectively. Development of novel and advanced detoxification processes to eliminate color and other inhibitors and feeding the fermenter low-cost nutrient sources can improve performance. The application of mixed microbial cultures and/or defined microbial consortia along with a co-substrate strategy using a pretreated fraction of SCGO to optimize carbon utilization and increase overall PHA production, should also be explored. These approaches could further strengthen the viability of developing sustainable SCGO-based biorefineries for the sustainable production of biodegradable bioplastics.

### 3.4. Characterization of Produced Polyhydroxyalkanoate (PHA)

#### 3.4.1. X-Ray Diffraction (XRD) Analysis

**Figure 5:**
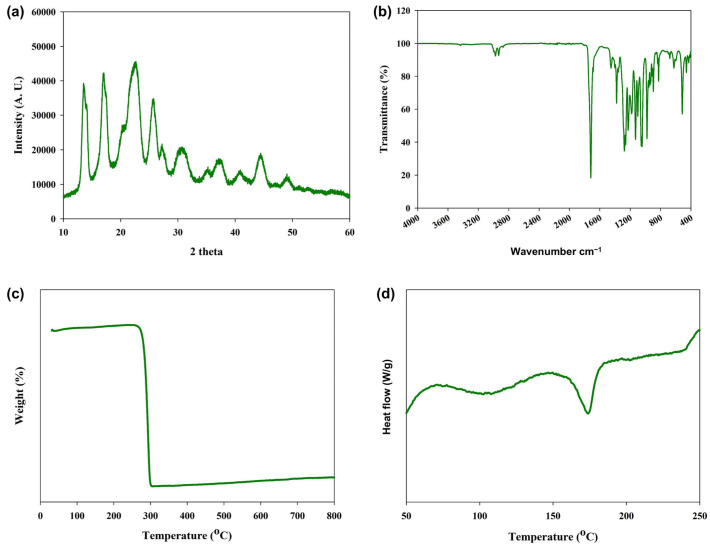
(**a**) XRD, (**b**) FT-IR, (**c**) TGA and (**d**) DSC analysis of PHA produced by *Lysinibacillus* sp. RG.S. using SAEAc-SCGO hydrolysates (20 g/dm^3^).

The crystal structure of extracted PHA was examined by means of X-ray diffraction (XRD). The prominent diffraction peaks at 2θ angles of 13.64° and 16.88° revealed the presence of a well-defined crystalline phase inside the polymer matrix (Figure 5a). The formation of a highly ordered polymeric arrangement is further verified by additional intense reflections at 21.58° and 23.15°, which are characteristic of the orthorhombic crystal lattice structure inherent in the α-form PHB crystal. Additionally, the presence of diffraction peaks at 25.85° and 27.56° confirms the presence of partially crystalline domains, characteristic of PHB synthesized by biological organisms. These characteristic diffraction peaks indicate a semicrystalline structure with a high crystallinity in the extracted polymer, which accounts for its mechanical strength, heat resistance, and molding ability. The diffraction patterns and crystal structure found were similar to the previous reports on PHB synthesized by various microbial systems, indicating that the polymer synthesized herein is structurally intact and exhibits crystallographic characteristics [36,60,61].

#### 3.4.2. Fourier Transform Infrared Spectroscopy (FTIR) Analysis

The FTIR spectrum shown in Figure 5b displayed a number of characteristic bands of the PHA and was representative of a successful biosynthesis of this polyester. Absorption peaks at 2986 cm^−1^ and 2987 cm^−1^ are attributed to the asymmetric and symmetric stretch of C–H bonds in the polymer backbone of aliphatic groups such as CH_2_ and CH_3_ [62]. An important absorption band in the spectrum was the very strong band at 1718 cm^−1^, corresponding to the stretching vibration of the ester carbonyl (C=O), a characteristic feature of PHB and other polyhydroxyalkanoates [36]. The absorption band found around 1286 cm^−1^ was assigned to deformation and rotational vibrations of –CH groups [63]. The bands at 1462 cm^−1^ and 1382 cm^−1^ were attributed to bending of –CH_2_ and –CH_3_ groups, respectively [60]. Furthermore, the strong absorption at 1042 cm^−1^ is assigned to the stretching motion of C–O–C bonds that are linked to an ester group in the polymer backbone [64]. A few more bands in the fingerprint region (978–513 cm^−1^) can be assigned to the C–O and C–O–C stretching modes, further supporting the polyester nature of the extracted material. All together these characteristic absorption bands unambiguously confirm the molecular structure of PHB and demonstrate that the microbial strain has accumulated a highly pure polyester. The FTIR spectrum acquired in this study is also consistent with the previously reported spectra of PHB and microbially synthesized PHB [60,63,64,65]. It confirms the identification of the extracted biopolymer.

#### 3.4.3. Thermogravimetric (TGA) and Differential Scanning Calorimetry (DSC) Analysis

The thermal stability and melting properties of the fabricated PHB were extensively studied using thermal gravimetric analysis (TGA) and differential scanning calorimetry (DSC). TGA was performed to evaluate the ability of the polymer to withstand thermal degradation by measuring changes in sample mass against temperature. The typically high thermal degradation temperature (*T_d_*) of extracted PHB, approximately 272.5 °C, shown in Figure 5c, indicates that the extracted PHB is highly thermally stable. The significant weight loss at this temperature is likely due to the depolymerization and random scission of the ester bonds via a β-elimination mechanism, which is the main degradation mode of PHB.

Notably, there was no apparent inorganic ash left after thermal decomposition, reflecting the high inorganic contaminant purity of the recovered polymer. The behavior of thermal degradation obtained in the present study is in accordance with earlier reports including PHB synthesized using *Bacillus sphaericus* (NII 0838), for which a maximum degradation temperature was between 280 and 291 °C [66]. The DSC analysis was used to further explore the phase transitions of the polymer, and the thermogram is shown in Figure 5d. The melting temperature (*T_m_*) of the produced PHB was measured to be 175.5 °C, which agrees well with those reported for a standard PHB (~178 °C). The shape of the endotherm melting may offer further information on polymer structure, and a slight shoulder might suggest structural heterogeneity that should be investigated. These properties of relatively high melting point suggest that well-ordered crystalline domains are present in the polymer matrix and that the polymer has high degrees of regularity. Overall, TGA and DSC measurements show that the resulting PHB is thermally stable and crystalline. The results are also corroborated by XRD data, which revealed the characteristic α crystalline orthorhombic domains. The melting temperatures observed and those reported in the literature agree fairly well, which indicates high-quality, crystal clear homopolymer PHB, and suggests suitability for advanced biomaterial applications [36,60,67].

### 3.5. Phytochemical Profiling, Biosynthesis, Characterization of SCGO-Derived Silver Nanoparticles (SCGO-AgNPs)

The preliminary phytochemical screening of the solvent extractives derived from SCGO revealed numerous bioactive compounds such as, phenolic compounds, flavonoids, alkaloids, terpenoids, saponins, tannins, carbohydrates and cardiac glycosides (Table 3). Phytochemicals that were not detected during the qualitative analysis are not discussed further. The phenolic compounds were found to be abundant among the identified metabolites and have been involved in different biological activities such as antioxidant, antimicrobial, antidiabetic and anti-inflammatory properties. In a consistent agreement with the current study, Yang et al. (2018) and Firdaus et al. (2026) [49,50] described high levels of polyphenolic acids in the extracts obtained from spent coffee waste biomass. The total phenolic content (TPC) of SCGO extract was 2.15 mg GAE/g of SCGO, which is similar to previously reported values [50,51] and suggests a high concentration of phytochemicals with a redox activity. Moreover, flavonoid content is also higher, with a value of 1.10 ± 0.01 mg of QE/g of SCGO.

**Figure 6:**
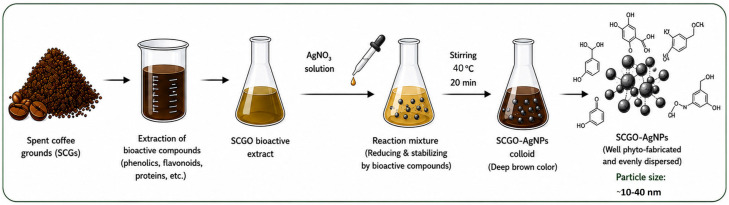
Schematic representation of the synthesis and stabilization mechanism of SCGO-AgNPs using SCGO solvent extractives.

The phytochemical reservoir was exploited in which the SCGO extract was used as both a reducing and a stabilizing agent for the one-pot green synthesis of AgNPs. Upon addition of the silver precursor solution a noticeable change in color from pale yellow to dark brown was observed within 20 min of incubation, signifying the reduction in Ag^+^ ions and the generation of silver nanoparticles. This phenomenon is well known and has been attributed to the surface plasmon resonance effect of the nanoscale silver, suggesting the reduction activity of bioactive compounds from the SCGO extract [68]. The phytochemicals not only facilitate nanoparticle formation but also act as capping and stabilizing agents, thereby inhibiting aggregation and augmenting colloidal stability (Figure 6). The phytochemical constituents when adsorbed onto the surface of the nanoparticles provide steric and electrostatic stabilization and may contribute to the long-term stability. These outcomes entail the capability of SCGO extract as an efficient and sustainable biogenic platform for rapid synthesis of stable silver nanoparticles that can be used in biomedical and antibacterial applications.

#### Structural Characterization of the Biosynthesized SCGO-AgNPs

**Figure 7:**
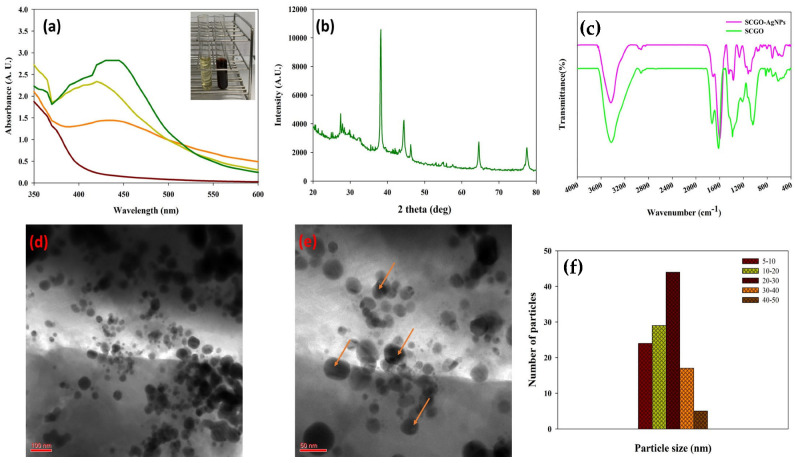
(**a**) UV-visible SPR spectrum at different incubation times, (**b**) X-ray diffraction pattern, (**c**) FTIR spectra, (**d**,**e**) HRTEM micrographs at 100 and 50 nm magnification, and (**f**) average particle size histogram of the synthesized SCGO-AgNPs.

Initially, silver nanoparticles (AgNPs) formed by SCGO were monitored by UV–Vis spectroscopy. When the silver precursor solution was mixed with the SCGO extract, the reaction mixture color turned reddish-brown within a few seconds, suggesting the reduction in Ag^+^ ions followed by the formation of silver nanoparticles. The UV–Vis spectrum displayed a broad SPR band with a peak at 445 nm (Figure 7a), which indicates spherical silver nanoparticles. The SPR intensity gradually increased with incubation duration, indicating continued formation of AgNPs, and plateaued after 20 min, indicating that all available silver ion was consumed and the synthesis of nanoparticles was stabilized. The absorption maximum exhibited here falls within the range of characteristic values reported for AgNPs fabrication with SCGO plant extracts as reducing agents [69,70]. X-ray diffraction (XRD) method was then used to analyze the crystallinity of the synthesized SCGO-AgNPs (Figure 7b). Four distinct peaks were observed in the diffraction pattern at scattering angles of 2θ = 38.30°, 46.23°, 65.11° and 77.56°, which can be attributed to the crystallographic planes (111), (200), (220) and (311) of the face-cantered cubic (fcc) structure of the silver material, respectively. Among the observed reflections, the (111) was found to be the strongest, suggesting preferential formation of crystal growth along the (111) orientation. The absence of other impurity peaks further supports the high phase purity of the biosynthesized nanoparticles. The diffraction pattern confirms the crystalline nature of the synthesized SCGO-AgNPs.

The FT-IR spectroscopy analysis of the SCGO extract and synthesized SCGO-AgNPs was conducted to elucidate the presence of functional groups in the synthesis and stabilization of the NPs (Figure 7c). The wide adsorption band at 3425 cm^−1^ was ascribed to O–H stretching vibrations of hydroxyl groups that are also associated with polyphenols and polysaccharides such as cellulose and hemicelluloses [71]. The peak at 1736 cm^−1^ was the most significant to C=O stretching vibrations of esterified phenolic compounds, e.g., chlorogenic, caffeic and *p*-coumaric acids, known to be present in the coffee biomass. A sharp peak at 1659 cm^−1^ was assigned to carbonyl groups related to the presence of caffeine and related aromatic structures [50,51]. The absorption signal at 1379 cm^−1^ indicated a symmetric C–H bending vibration of the aliphatic groups of constituents of coffee oil and the signal of 1032 cm^−1^ was assigned to the stretching vibration of C–O, C–C in the structures related to cellulose [49]. Aromatic ring vibrations and C–H deformation modes were assigned to additional bands found on the 864–588 cm^−1^ region.

The morphology and particle size distribution of the produced SCGO-AgNPs were assessed using the high-resolution TEM (HR-TEM). The micrographs showed spherically shaped and uniformly distributed nanoparticles with minimal aggregation. HR-TEM images at various magnifications (50 nm and 100 nm) indicated the well-defined nanostructure and homogeneous distribution of the particles (Figure 7d,e). The phytochemical constituents in the SCGO extract with their natural capping effect caused the formation of a thin organic layer surrounding the NP surface. Further particle size distribution analysis revealed that the majority of nanoparticles synthesized were in the range 10–40 nm (Figure 7f) signifying that the nanoparticles have a narrow size distribution. Particle size uniformity and size scales are beneficial for improving the physicochemical and biological activity of AgNPs. These observations agree with earlier reports of silver nanoparticle synthesis by plants [32]. The reflection spectrum is co-related with the standard values from the JCPDS card no. 04-0783.

### 3.6. Unlocking Multifunctional Potential of SCGO-AgNPs

**Figure 8:**
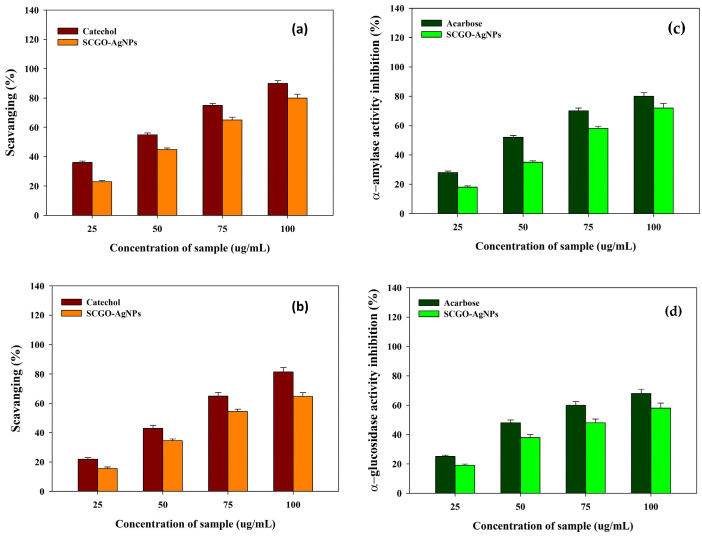
Antioxidant potential in terms of free radical scavenging activity against (**a**) DPPH and (**b**) ABTS, and antidiabetic potential in terms of inhibitory activity against (**c**) α-amylase and (**d**) α-glucosidase of synthesized SCGO-AgNPs.

The antioxidant assay was employed to assess the multi-potential effects of SCGO-AgNPs. SCGO-AgNPs exhibited considerable antioxidant activity (80.4%) at various concentrations (100 µg/mL) compared to the standard compound (catechol) (90.28%) for the DPPH free radical scavenging assay at the same concentrations (Figure 8a). The IC_50_ of SCGO-AgNPs was determined to be 62.95 µg/mL, while catechol showed an IC_50_ of 45.45 µg/mL. The DPPH Assay showed a higher scavenging capacity compared to the ABTS assay. For the ABTS scavenging assay, the scavenging property of the catechol was found to be better with 81.54% at 100 µg/mL and 58.45 µg/mL of IC_50_ compared to SCGO-AgNPs with 72.80% at 100 µg/mL and 84.50 µg/mL of IC_50_ (Figure 8b). Biosynthesized SCGO-AgNPs exhibited considerable antioxidant activity, indicating their high potential in scavenging reactive oxygen species (ROS) and thereby reducing oxidative stress. SCGO extract is rich in phenolic compounds, flavonoids, and other bioactive metabolites which can reduce Ag^+^ ions during their formation and act as stabilizing and capping agents on the surface of nanoparticles [51]. Surface-based phytochemicals particularly phenols are known to exhibit strong electron donating and hydrogen transfer properties, leading to effective free radical quenching and ROS scavenging [51]. All of these results confirm that the phytochemicals coating the surface of the SCGO-AgNPs is critical for enhancing their antioxidant activity, making biogenic surface functionalization crucial for the production of a multifunctional nanomaterial with increased biological activity.

Hyperglycemia can be regulated by inhibiting carbohydrate catalyzing enzymes, such as α-amylase, which could prevent the hydrolysis of complex sugars into monosaccharides, leading to elevated blood sugar [72,73].

α-Amylase breaks down complex starch into simple glucose [74]. Carbohydrate digestion is mainly carried out by pancreatic α-amylase and intestinal α-glucosidase which break down complex carbohydrates and oligosaccharides into absorbable carbohydrates that are monosaccharides. Inhibition of these enzymes is a proven therapeutic approach for type 2 diabetes mellitus by slowing the breakdown of carbohydrates thereby reducing glucose absorption and postprandial hyperglycemia. Thus, new inhibitor agents of carbohydrate hydrolyzing enzymes have been the focus of many studies aimed at developing an effective antidiabetic therapy. The antidiabetic effect of SCGO-AgNPs was then assessed for α-amylase and α-glucosidase (Figure 8d). In comparison, SCGO-AgNPs exhibited strong and positive 72.12% and 58.42% while acarbose exhibited a very good 80.15% and 68.45% inhibition for α-amylase and α-glucosidase inhibition at the tested concentration (100 µg/mL) (Figure 8c,d). The IC_50_ values of SCGO-AgNPs were 70.41 and 80.54 μg/mL for α-amylase and α-glucosidase inhibition, respectively while, those of acarbose were 54.45 and 61.45 μg/mL, respectively. The present study showed that the SCGO-AgNPs showed higher inhibitory activity than acarbose against both α-amylase and α-glucosidase, indicating that they could be used as nanomaterials in the pharmaceutical industry for antidiabetic applications. By inhibiting vital digestive enzymes, SCGO-AgNPs may effectively slow the conversion of some dietary starches into glucose, therefore aiding blood sugar control and reducing postprandial blood glucose elevation [72]. The remarkable enzyme inhibitory property of SCGO-AgNPs is probably ascribed to the binding of phytochemicals, phenolics, flavonoids, or other bioactive metabolites in the SCGO extract (chelate) to the AgNPs surface during the biosynthetic process. Altogether, it is concluded that due to their strong inhibitory effect on carbohydrate-digesting enzymes and on glucose regulation following carbohydrate intake, these results may suggest therapeutic applications of SCGO-AgNPs as multifunctional nanobiomaterials for diabetes management.

## 4. Conclusions

This study introduces an integrated cascading biorefinery strategy efficiently and comprehensively convert defatted spent coffees (SCGO) into high-value-added products. The results of the combined solvent extraction–acid pretreatment–enzymatic hydrolysis–activated carbon detoxification (SAEAc) technique showed excellent performance in removing fermentation inhibitors and significantly improving the fermentability of the hydrolysate, which resulted in better biomass growth and a higher polyhydroxyalkanoate (PHA) yield in *Lysinibacillus* sp. RG.S. The PHA obtained showed physicochemical and thermal properties similar to those of commercial PHB, indicating the potential of SCGs as a sustainable feedstock for bioplastic production. Simultaneously, bioactive compounds extracted from SCGs enabled the green synthesis of uniformly dispersed silver nanoparticles that showed remarkable antioxidant, and antidiabetic activities. The production of such biopolymer and nanomaterials on a single platform enables zero-waste biorefining and provides a scalable approach for converting coffee processing waste into commercially relevant biomaterials. The results demonstrate the potential of an integrated SCG biorefinery platform for advancing circular bioeconomy principles, waste valorization, and the sustainable production of valuable products.

## Figures and Tables

**Table 1:** Chemical composition of spent coffee grounds (SCG) used in this study (g/100 g of dry SCGs).

Name of Component	Chemical Composition (%)
Dry solid matter	96.2 ± 2.15
Proteins	8.50 ± 0.44
Fats/Lipids	11.5 ± 1.14
Lignin	20.9 ± 1.15
Total sugars	49.40 ± 2.24
Cellobiose	2.60 ± 0.08
Glucose	6.35 ± 0.32
Mannose	17.25 ± 0.41
Galactose	20.20 ± 0.48
Arabinose	0.80 ± 0.001
Xylose	2.25 ± 0.01

Values are the mean of the three performed experiments; (±) standard error; (SE) by one-way ANOVA with Tukey–Kramer multiple comparisons test.

**Table 2:** Effects of various detoxification strategies on SCGO feedstock (20 g/dm^3^) and their effects on assimilation of sugar, *Lysinibacillus* sp. RG.S. growth and PHA production parameters.

Type of SCGO Detoxification Process	Total Sugar Utilization (%)	Dry Cell Weight (DCW, g/dm^3^)	PHA Accumulation (%)	PHA Titer (g/dm^3^)
SCGO-AE	30.0 ± 1.45	2.82 ± 0.10	40.2 ± 2.15	1.12 ± 0.01
SCGO-SAE	38.2 ± 1.89	3.57 ± 0.16	45.0 ± 2.20	1.60 ± 0.02
SCGO-SAEAc	50.0 ± 2.52	4.90 ± 0.20	48.1 ± 2.15	2.36 ± 0.02

Values are the mean of three experiments; (±) standard error; (SE) by one-way ANOVA with Tukey–Kramer multiple comparisons test.

**Table 3:** Determination of various bioactive compounds and total phenolic concentration of SCGO.

Name of Phytochemicals	Empty Cell
Tannins	+
Alkaloids	+
Terpenoids	+
Steroids	−
Saponins	+
Cardiac steroidal glycosides	+
Phenols	+
Anthraquinones	+
Total polyphenols (TPC) *	2.15 ± 0.01 (mg of GAE/g of SCGO)
Total flavonoids (TFC) *	1.10 ± 0.01 (mg of QE/g of SCGO)

(+) means Present and (−) means Absent. * Values are means of three experiments ± SE.

## Data Availability

The raw data supporting the conclusions of this article will be made available by the authors on request.
